# The role of adjuvant chemotherapy in rectal cancer: A nationwide cohort study from the Netherlands

**DOI:** 10.1111/codi.70054

**Published:** 2025-03-09

**Authors:** Aaya Darai, Tijmen Koëter, Felice N. van Erning, Robbert J. van Alphen, Henk M. W. Verheul, Marcel Verheij, David D. E. Zimmerman, Pauline A. J. Vissers, Johannes H. W. de Wilt

**Affiliations:** ^1^ Department of Surgery Radboud University Medical Center Nijmegen The Netherlands; ^2^ Department of Surgery Elisabeth TweeSteden Hospital Tilburg The Netherlands; ^3^ Department of Research and Development Netherlands Comprehensive Cancer Organization (IKNL) Utrecht The Netherlands; ^4^ Department of Surgery Catharina Hospital Eindhoven The Netherlands; ^5^ Department of Oncology Elisabeth TweeSteden Hospital Tilburg The Netherlands; ^6^ Department of Oncology Radboud University Medical Center Nijmegen The Netherlands; ^7^ Department of Radiation Oncology Radboud University Medical Center Nijmegen The Netherlands

**Keywords:** chemotherapy, rectal cancer, rectal surgery, systemic therapy

## Abstract

**Aim:**

Treatment of rectal cancer has improved significantly over the past decades. However, the role of adjuvant chemotherapy remains a matter of debate. The aim of this study is to evaluate the association between adjuvant chemotherapy and overall survival of patients with rectal cancer.

**Method:**

Data from the Netherlands Cancer Registry were used to evaluate all nonmetastatic pathological node‐positive patients who underwent treatment for rectal cancer during the time period 2009–2020 in the Netherlands. Patients were grouped according to whether they received adjuvant chemotherapy. Patients were further divided into three groups according to the type of preoperative treatment. Propensity score matching (PSM) was applied based on patient‐related variables, tumour‐related variables and treatment‐related variables. The matching procedure for PSM was done with nearest neighbour and without replacement employing a 1:1 ratio. Kaplan–Meier analysis was performed after PSM to compare overall survival.

**Results:**

A total of 7479 patients were included, of whom 865 (11.6%) underwent adjuvant chemotherapy. After PSM the no neoadjuvant treatment group included 240 patients per study arm, the neoadjuvant radiotherapy group 317 and the neoadjuvant chemoradiation group 182 patients. A significant difference in 5‐year survival was found comparing adjuvant versus no adjuvant chemotherapy in all subgroups: no neoadjuvant treatment 54.6% vs. 40.8% (*p* = 0.003), neoadjuvant radiotherapy 77.0% vs. 53.9% (*p* < 0.001) and neoadjuvant chemoradiation 68.1% vs. 45.6% (*p* < 0.001).

**Conclusion:**

Adjuvant chemotherapy was associated with an improved 5‐year survival in all subgroups. The role of adjuvant chemotherapy in the treatment of rectal cancer should be reconsidered in node‐positive patients.

**What does this paper add to the literature?**

The role of adjuvant chemotherapy in the treatment of rectal cancer remains a matter of debate. This study investigates the impact of adjuvant chemotherapy on overall survival in patients with nonmetastatic rectal cancer using Netherlands Cancer Registry data. Adjuvant chemotherapy significantly improved 5‐year survival across different preoperative treatment groups. This study suggests a reconsideration of adjuvant chemotherapy in rectal cancer treatment, highlighting its potential to enhance survival outcomes, and addresses ongoing debates about its role in patient care.

## INTRODUCTION

Adjuvant chemotherapy for colon cancer reduces disease recurrence and improves survival and has become standard treatment for patients with high‐risk Stage II–III colon cancer [1, 2, 3]. It usually consists of capecitabine and oxaliplatin (CAPOX) or 5‐fluorouracil, leucovorin and oxaliplatin (FOLFOX), which preferably starts within 4–8 weeks after surgery [4, 5, 6]. Although rectal cancer has histological similarities with colon cancer [7], there are substantial differences in the approach to how we deal with rectal cancer compared with colon cancer, such as the use of preoperative high‐quality MRI, neoadjuvant (chemo)radiotherapy regimens and total mesorectal excision (TME)—the standard surgical technique. Currently, there is no global consensus towards adjuvant chemotherapy treatment in rectal cancer; for example, the National Comprehensive Cancer Network in the USA recommends adjuvant chemotherapy in the treatment of Stage III rectal cancer [8]. However, the European Society for Medical Oncology (ESMO) considers the level of scientific evidence low and thus recommends that the decision on adjuvant chemotherapy should be jointly made by the patient and clinician [9, 10].

A Cochrane systematic review showed that rectal cancer patients who were not treated with neoadjuvant (chemo)radiotherapy benefitted from adjuvant chemotherapy in terms of overall survival (OS) and disease‐free survival (DFS) [11]. However, a meta‐analysis of four trials showed no improvement in OS or DFS [12]. Although adjuvant chemotherapy in rectal cancer is not recommended by the Dutch guidelines [13], a recently published survey showed that some medical oncologists in the Netherlands do offer adjuvant chemotherapy to patients with Stage III rectal cancer [14].

The current understanding of the efficacy of adjuvant chemotherapy in the management of nonmetastatic rectal cancer reveals a discernible knowledge gap pertaining to the impact of adjuvant chemotherapy on survival. Recently, a retrospective study was published regarding the survival benefit in clinically node‐negative but pathologically node‐positive patients treated with adjuvant chemotherapy. The study focused only on patients who had not received any preoperative treatment. However, as neoadjuvant treatment is recommended in a substantial group of patients with rectal cancer, this patient group should be included in these types of analyses [15].

In this present study we assessed the association between adjuvant chemotherapy and OS in patients who either underwent neoadjuvant treatment (radiotherapy or chemoradiotherapy) or received no neoadjuvant treatment, using data from a nationwide database.

## METHOD

### Study design and data collection

Data were retrieved from the nationwide population‐based Netherlands Cancer Registry (NCR). The NCR is notified by the nationwide registry of histo‐ and cytopathology (PALGA) [16] of all newly diagnosed cancer patients in the Netherlands. Trained data managers routinely extract data on patient and tumour characteristics, diagnosis and treatment from medical records in all hospitals in the Netherlands. Survival time could be computed by the annual linkage of the NCR with data from the nationwide Personal Records Database, containing information on vital status and date of death for all Dutch inhabitants. These data were complete up to 1 February 2023. Tumour stage was recorded according to the International Union Against Cancer TNM classification (2009, 6th edition; 2010–2016, 7th edition; 2017 onwards, 8th edition). There were no relevant differences between the abovementioned TNM classifications for rectal cancer. The study was designed and reported in accordance with the STROBE checklist to ensure standardized reporting. This study was approved by the Privacy Review Board of the NCR and the scientific committee of the Prospective Dutch ColoRectal Cancer cohort. According to Dutch and European law the study did not require approval from an ethics committee in the Netherlands.

### Patients

All patients diagnosed between 2009 and 2020 and undergoing surgery for non‐metastatic rectal cancer with a pathological nodal stage of 1 or 2, were evaluated. The following patient‐ and tumour‐related variables were available: age, sex, vital status (alive or deceased) and pathological tumour and nodal stage. Other available treatment‐related variables were year of diagnosis, type of surgical procedure (low anterior resection or abdominoperineal resection), type of neoadjuvant treatment and perioperative complications (specifically the occurrence of anastomotic leakage and abscess).

Patients who had an unknown or missing status in any of the abovementioned variables were excluded from analysis in order to adequately match the groups during propensity score matching (PSM). Patients who were treated with only neoadjuvant chemotherapy were also excluded from analysis due to low patient numbers. All patients who died in the first 6 months after diagnosis (*n* = 449, 5.7%) were excluded from analysis in order to correct for the treatment period. This included 193 who did not undergo any neoadjuvant treatment, 220 who underwent radiotherapy and 36 who underwent chemoradiation.

### Statistical analysis

Baseline characteristics were described and reported as median (interquartile range, IQR) for continuous data and frequency (percentage) for categorical data. Trend analysis was performed using the Cochran–Armitage test. Differences between the adjuvant chemotherapy group versus the control group were assessed with a chi‐square test or independent *t*‐test. For the survival analysis the follow‐up time was calculated from 6 months after diagnosis until date of death or the end of the follow‐up (1 February 2023). Kaplan–Meier analyses were performed to present survival data.

### Propensity score matching

There were three patient subgroups (i.e. no neoadjuvant treatment, neoadjuvant radiotherapy, neoadjuvant chemoradiotherapy). The type of neoadjuvant radiotherapy was not available in the NCR for the majority of the study period, but according to the Dutch guidelines consisted of 5× 5 Gy short‐course radiotherapy in 1 week. Chemoradiation usually consists of 25× 2 Gy short‐course radiotherapy with concomitant capecitabine according to our guidelines [17]. PSM was used according to a logistic regression model to create two equally balanced groups for adjuvant chemotherapy versus no adjuvant chemotherapy. The following case‐mix variables were addressed: patient‐related variables (age, gender, year of diagnosis), tumour‐related variables (pathological tumour and nodal stage) and treatment‐related variables (type of surgical procedure, neoadjuvant treatment, anastomotic leakage). The matching procedure for PSM was done with nearest neighbour and without replacement employing a 1:1 ratio. The chi‐square test was used to check whether the groups were balanced before PSM. After PSM, standardized mean difference (SMD) was calculated to assess covariate balance between groups after PSM. Comparison between the two groups after PSM was done with log‐rank tests.

### Outcomes

The primary objective of this study was to assess overall 5‐year survival rates as the primary outcome measure. Kaplan–Meier curves were employed to analyse these survival outcomes.

A *p*‐value of <0.05 was considered statistically significant. A SMD < 0.1 was considered to indicate sufficient balance. Analyses were performed in SPSS 28.0 Statistics for Windows (IBM Corp., Armonk, NY).

## RESULTS

**FIGURE 1:**
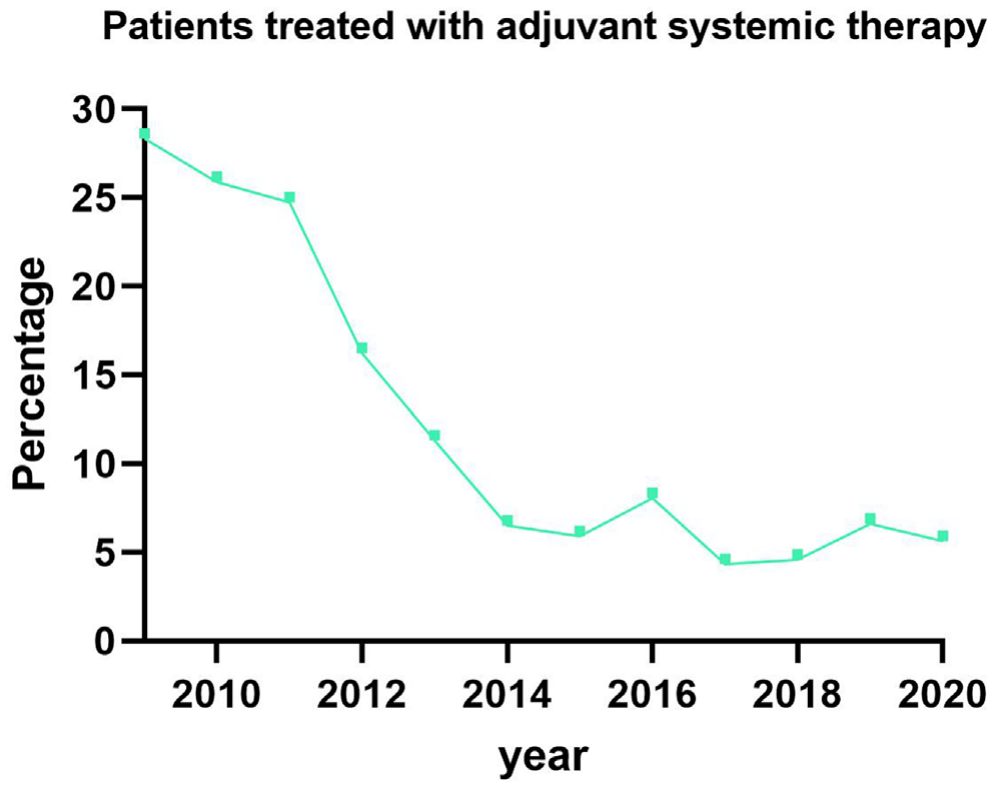
Percentage of rectal patients who received adjuvant treatment over the years.

The baseline characteristics of all patients are shown in Table S1. A total of 7479 patients who underwent surgery for rectal cancer in the period between 2009 and 2020 and were diagnosed with pathologically positive nodal disease [(y)pN1, *n* = 5549; (y)pN2, *n* = 1930] were included, as shown in supplementary flowchart 1. Of these, 865 patients were treated with adjuvant chemotherapy (11.6%). Most patients (*n* = 744, 86.0%) received adjuvant treatment before 2017, showing a significant decrease in adjuvant chemotherapy over the years in the Netherlands (Figure 1; *p* < 0.001). A total of 1966 patients did not receive any neoadjuvant treatment, of whom 319 did receive adjuvant chemotherapy. A total of 3020 patients were treated with neoadjuvant radiotherapy, of whom 356 received adjuvant chemotherapy. The other 2493 patients received neoadjuvant chemoradiation, of whom 190 received adjuvant chemotherapy.

### Patients with rectal cancer without neoadjuvant treatment

The baseline characteristics of the patients who did not undergo neoadjuvant treatment (*n* = 1966) are shown in Table 1. A total of 319 (16.2%) patients underwent adjuvant chemotherapy. After PSM, 240 of 319 (75.2%) patients who underwent adjuvant chemotherapy could be matched to 240 patients without adjuvant therapy. In the PSM sample there were no significant differences between the two groups.

**TABLE 1:** Patient, tumour and treatment characteristics of rectal cancer patients who were not treated with neoadjuvant therapy, before and after propensity score matching (PSM).

		Before PSM			After PSM		
	Group	No adjuvant systemic therapy (*N* = 1647, *n* (%)	Adjuvant systemic therapy (*N* = 319), *n* (%)	SMD	No adjuvant systemic therapy (*N* = 240), *n* (%)	Adjuvant systemic therapy (*N* = 240), *n* (%)	SMD
Patient characteristics							
Age (years)				0.563			0
	<60	324 (19.7)	112 (35.1)		72 (30.0)	72 (30.0)	
	61–70	540 (32.8)	135 (42.3)		104 (43.3)	104 (43.3)	
	71–80	541 (32.8)	65 (20.4)		58 (24.2)	58 (24.2)	
	>80 yrs	242 (14.7)	7 (2.2)		6 (2.5)	6 (2.5)	
Sex				−0.062			
	Male	1028 (62.4)	189 (59.2)		156 (65.0)	156 (65.0)	
	Female	619 (37.6)	130 (40.8)		84 (35.0)	84 (35.0)	
Year of diagnosis				0.577			
	2009–2012	158 (9.6)	99 (31.0)		52 (21.7)	52 (21.7)	
	2013–2016	708 (43.0)	125 (39.2)		107 (44.6)	107 (44.6)	
	2017–2020	781 (47.4)	95 (29.8)		81 (33.8)	81 (33.8)	
Tumour characteristics							
(y)pT stage				−0.300			
	(y)pT0– (y)pT2	663 (40.3)	90 (28.2)		71 (29,6)	71 (29,6)	
	(y)pT3	914 (55.6)	191 (60.1)		155 (64.6)	155 (64.6)	
	(y)pT4	68 (4.1)	37 (11.6)		14 (5.8)	14 (5.8)	
(y)pN stage				−0.418			
	(y)pN1	1339 (81.3)	203 (63.6)		159 (66.3)	159 (66.3)	
	(y)pN2	308 (18.7)	116 (36.4)		81 (33.8)	81 (33.8)	
Treatment characteristics							
Type of surgery				0.386			
	(Low) anterior resection	1347 (81.8)	305 (95.6)		229 (95.4)	229 (95.4)	
	Abdominoperineal resection	300 (18.2)	14 (4.4)		11 (4.6)	11 (4.6)	
Anastomotic complications				0.444			
	No leakage or abscess	1082 (65.7)	272 (85.3)		145 (60.4)	145 (60.4)	
	Leakage and/or abscess	169 (10.3)	25 (7.8)		61 (8.2)	61 (8.2)	
	Not applicable^a^	396 (24.0)	22 (6.9)		34 (4.6)	34 (4.6)	

Abbreviation: SMD, standardized mean difference.

^a^ Not applicable due to having a deviating stoma.

**FIGURE 2:**
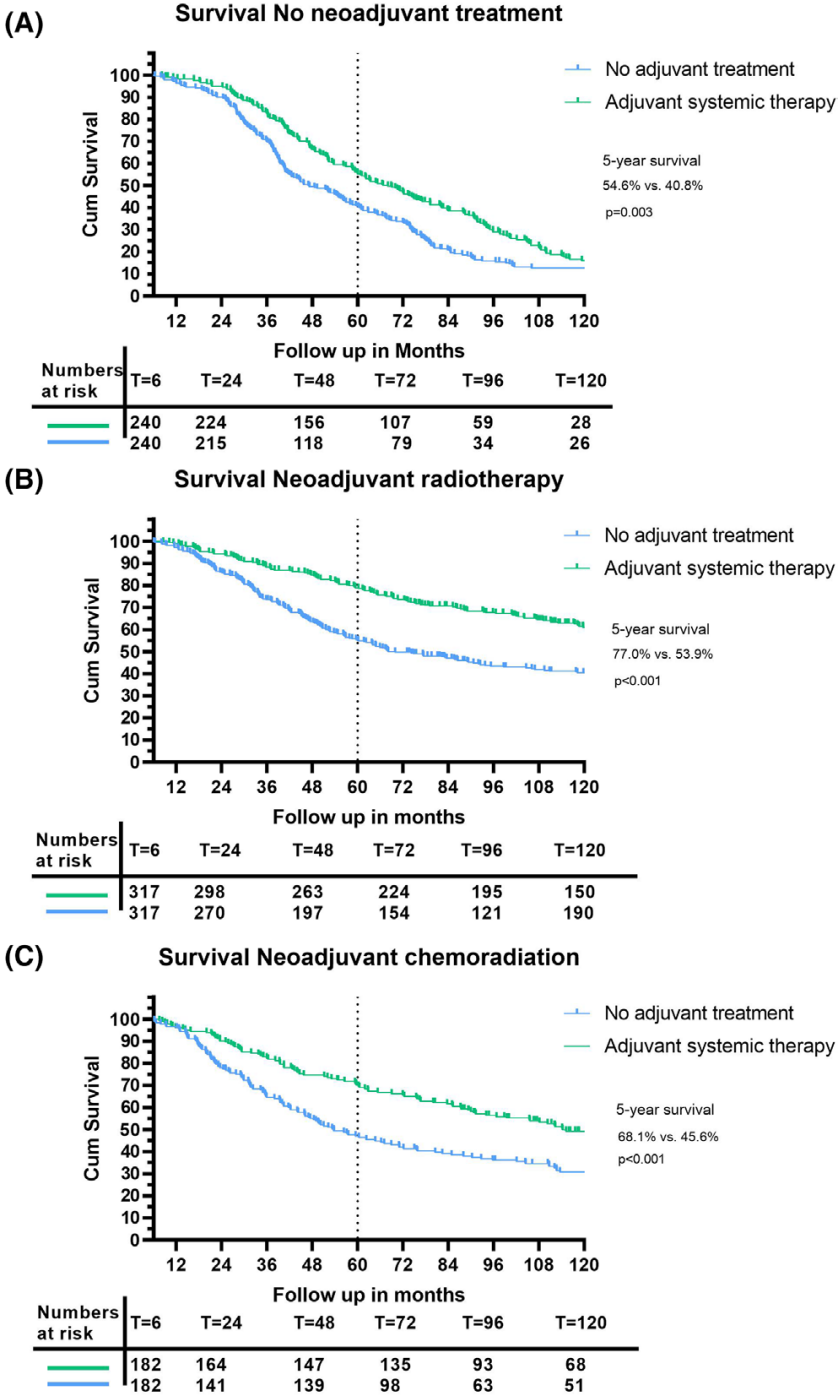
Kaplan–Meier curve for rectal cancer patients who (A) were not treated with neoadjuvant therapy, (B) were treated with neoadjuvant radiotherapy or (C) neoadjuvant chemoradiotherapy after propensity score matching comparing adjuvant systemic therapy versus the control group.

Median follow‐up was 65 months (IQR 57–73 months) in the adjuvant chemotherapy group and 47 months (IQR 39–55 months) in the control group. Figure 2A shows the survival curves of both groups after PSM. The OS rate was significantly greater in the patients treated with adjuvant chemotherapy. The 5‐year OS rate was respectively 54.6% (95% CI 48.3%–60.9%) versus 40.8% (95% CI 34.6%–47.1%) (*p* = 0.003).

### Patients with rectal cancer who underwent neoadjuvant radiotherapy

The baseline characteristics of the patients who underwent neoadjuvant radiotherapy (*n* = 3020) are shown in Table 2. A total of 356 (12.1%) patients with rectal cancer underwent adjuvant chemotherapy. After PSM, 317 of the 356 (86.8%) patients remained for further analyses without significant differences between the adjuvant versus no adjuvant chemotherapy groups.

**TABLE 2:** Patient, tumour, and treatment characteristics of rectal cancer patients who were treated with neoadjuvant radiotherapy, before and after propensity score matching (PSM).

	Group	Before PSM			After PSM		
		No adjuvant systemic therapy (*N* =2664), *n* (%)	Adjuvant systemic therapy (*N* = 356), *n* (%)	SMD	No adjuvant systemic therapy (*N* = 317), *n* (%)	Adjuvant systemic therapy (*N* = 317), *n* (%)	SMD

Patient characteristics							
Age (years)				0.711			0
	<60	595 (22.3)	169 (47.5)		139 (43.8)	139 (43.8)	
	61–70	904 (33.9)	134 (37.6)		129 (40.7)	129 (40.7)	
	71–80	848 (31.8)	53 (14.9)		49 (15.5)	49 (15.5)	
	>80	317 (11.9)	0				
Sex				0.021			
	Male	1679 (63.0)	229 (64.3)		204 (64.4)	204 (64.4)	
	Female	985 (37.0)	127 (35.7)		113 (35.6)	113 (35.6)	
Year of diagnosis				0.837			
	2009–2012	908 (34.1)	270 (75.8)		243 (76.7)	243 (76.7)	
	2013–2016	1049 (39.4)	68 (19.1)		61 (19.2)	61 (19.2)	
	2017–2020	707 (26.5)	18 (5.1)		13 (4.1)	13 (4.1)	
Tumour characteristics							
(y)pT stage				−0.125			
	(y)pT0– (y)pT2	818 (30.7)	93 (26.1)		84 (25.5)	84 (25.5)	
	(y)pT3	1753 (65.9)	236 (66.9)		221 (69.7)	221 (69.7)	
	(y)pT4	88 (3.3)	24 (6.8)		12 (3.8)	12 (3.8)	
(y)pN stage				−0.402			
	(y)pN1	1962 (73.6)	199 (55.9)		197 (62.1)	197 (62.1)	
	(y)pN2	702 (26.4)	157 (44.1)		120 (37.9)	120 (37.9)	
Treatment characteristics							
Type of surgery				0.262			
	(Low) anterior resection	2012 (75.5)	309 (86.8)		279 (88.0)	279 (88.0)	
	Abdominoperineal resection	652 (24.5)	47 (13.2)		38 (12.0)	38 (12.0)	
Anastomotic complications				0.470			
	No leakage or abscess	1795 (67.4)	305 (85.7)		274 (86.4)	274 (86.4)	
	Leakage and/or abscess	347 (13.0)	44 (12.4)		38 (12.0)	38 (12.0)	
	Not applicable^a^	522 (19.6)	7 (2.0)		5 (1.6)	5 (1.6)	

Abbreviation: SMD, standardized mean difference.

^a^ Not applicable due to having a deviating stoma.

Median follow‐up was 104 months (IQR 110–124 months) in the adjuvant group and 80 months (IQR 55–80 months) in the group not treated with adjuvant therapy. Figure 2B shows the survival curves for both groups. The OS rate was significantly greater in the patients treated with adjuvant chemotherapy. The 5‐year OS rate was, respectively, 77.0% (95% CI 72.3%–81.6%) versus 53.9% (95% CI 48.4%–59.4%) (*p* < 0.001).

### Patients with rectal cancer who underwent neoadjuvant chemoradiotherapy

Table 3 shows the baseline characteristics for rectal cancer patients who underwent neoadjuvant chemoradiotherapy (*n* = 2493), of whom 190 (7.6%) were treated with adjuvant chemotherapy. After PSM, 182 of 190 (95.8%) patients were well balanced without significant differences between the two groups.

**TABLE 3:** Patient, tumour and treatment characteristics of rectal cancer patients who were treated with neoadjuvant chemoradiotherapy, before and after propensity score matching (PSM).

	Group	Before PSM			After PSM		
		No adjuvant systemic therapy (*N* = 2303), *n* (%)	Adjuvant systemic therapy (*N* = 190), *n* (%)	SMD	No adjuvant systemic therapy (*N* = 182), *n* (%)	Adjuvant systemic therapy (*N* = 182), *n* (%)	SMD

Patient characteristics							
Age (years)				0.347			0
	<60	932 (40.5)	106 (55.8)		101 (55.5)	101 (55.5)	
	61–70	800 (34.7)	57 (30.0)		56 (30.8)	56 (30.8)	
	71–80	512 (22.2)	27 (14.2)		25 (13.7)	25 (13.7)	
	>80	59 (2.6)	0				
Sex				0.123			
	Male	1405 (61.0)	128 (67.4)		124 (68.1)	124 (68.1)	
	Female	898 (39.0)	62 (32.6)		58 (31.9)	58 (31.9)	
Year of diagnosis				1.026			
	2009–2012	580 (25.2)	141 (74.2)		136 (74.7)	136 (74.7)	
	2013–2016	1023 (44.4)	41 (21.6)		40 (22.0)	40 (22.0)	
	2017–2020	700 (30.4)	8 (4.2)		6 (3.3)	6 (3.3)	
Tumour characteristics							
(y)pT stage				–0.052			
	(y)pT0– (y)pT2	646 (28.1)	47 (24.7)		43 (23.6)	43 (23.6)	
	(y)pT3	1511 (65.6)	134 (70.5)		132 (72.5)	132 (72.5)	
	(y)pT4	146 (6.3)	9 (4.7)		7 (3.8)	7 (3.8)	
(y)pN stage				−0.366			
	(y)pN1	1733 (75.2)	113 (59.5)		113 (62.1)	113 (62.1)	
	(y)pN2	570 (24.8)	77 (40.5)		69 (37.9)	69 (37.9)	
Treatment characteristics							
Type of surgery				0.209			
	(Low) anterior resection	1461 (63.4)	138 (72.6)		131 (72.0)	131 (72.0)	
	Abdominoperineal resection	842 (36.6)	52 (27.4)		51 (28.0)	51 (28.0)	
Anastomotic complications				0.546			
	No leakage or abscess	1408 (61.1)	165 (86.8)		162 (89.0)	162 (89.0)	
	Leakage and/or abscess	258 (11.2)	12 (6.3)		9 (4.9)	9 (4.9)	
	Not applicable^a^	637 (27.7)	13 (6.8)		11 (6.0)	11 (6.0)	

Abbreviation: SMD, standardized mean difference.

^a^ Not applicable due to having a deviating stoma.

The median follow‐up was 99 months (IQR 86–111 months) in the adjuvant group and 51 months (IQR 41–61 months) in the group who were not treated with adjuvant therapy. Figure 2C shows the survival curves for both groups. The OS rate was significantly greater in the patients treated with adjuvant chemotherapy. The 5‐year survival rate was respectively 68.1% (95% CI 61.3%–74.9%) versus 45.6% (95% CI 38.3%–52.9%) (*p* < 0.001).

## DISCUSSION

In this retrospective cohort study of a Dutch nationwide registry the impact of adjuvant chemotherapy in the treatment of patients with nonmetastatic rectal cancer was investigated. A significant 5‐year survival benefit was demonstrated in all patient subgroups, regardless of preoperative treatment (neoadjuvant radiotherapy, chemoradiation or no neoadjuvant treatment). The role of adjuvant chemotherapy in rectal cancer patients has been an ongoing matter of debate, unlike in colon cancer where adjuvant chemotherapy is considered standard treatment in high‐risk Stage II and III disease [18, 19, 20]. A few European randomized controlled trials (RCTs) have been performed in the past, all showing no significant difference in survival in patients treated with neoadjuvant therapy [21, 22, 23]. Of these, however, the PROCTOR‐SCRIPT trial closed prematurely because of slow patient accrual. A meta‐analysis of this trial, together with three other randomized phase II studies [23, 24, 25], showed no improvement in OS or DFS [12]. Examining the results of the EORTC 22921 randomized study, less than 43% of the patients in the adjuvant chemotherapy group received the full treatment in the prescribed timeframe; therefore, conclusions regarding OS and DFS could be considered unreliable.

More recently, the results of the international, multicentre phase III RAPIDO trial also failed to demonstrate a difference in disease‐related treatment failure, but several remarks need to be made. First, patients were not randomized for treatment with or without adjuvant chemotherapy after previous chemoradiation therapy or short‐course radiotherapy followed by systemic CAPOX or FOLFOX. Second, it should be considered that the benefit of adjuvant chemotherapy was not a primary endpoint. Third, the study was not powered for this specific aspect [26].

In a Cochrane review from 2012, data were analysed from 21 RCTs comparing adjuvant chemotherapy versus observation in rectal cancer patients who were not treated with neoadjuvant (chemo)radiotherapy. In this review the investigators described that 5‐fluorouracil‐based postoperative chemotherapy improved OS by 17% and DFS by 25% [11]. In all trials patients were treated with radical rectal surgery, preceded or not by preoperative radiotherapy and randomized for adjuvant chemotherapy. A limitation of these trials was that the role of neoadjuvant treatment was not addressed, while neoadjuvant therapy is standard care for many rectal cancer patients.

Comparison of previously mentioned clinical studies yields no definitive conclusion regarding the role of adjuvant chemotherapy. However, adjuvant chemotherapy is still advised in many US and European guidelines. Currently, the ESMO recommends fluoropyrimidine alone or combined with oxaliplatin when considering adjuvant chemotherapy in Stage III rectal cancer [27], demonstrating the requirement for additional research regarding the value of adjuvant chemotherapy after neoadjuvant treatment and surgery.

The strength of this study is that we used a PSM to balance patient‐, tumour‐ and treatment‐related confounders. Fortunately, PSM gave approximately 75%–96% of the patients who underwent adjuvant chemotherapy a match in our three PSMs. The fact that we used anastomotic leakage as a variable in the propensity score analysis further strengthens the study. Anastomotic complications are associated with poor survival rates in both rectal and colon cancer [28, 29, 30]. Furthermore, the occurrence of anastomotic complications may reduce the likelihood of a patient receiving adjuvant chemotherapy, as these complications can lead to a less favourable clinical condition. We also included year of diagnosis in the PSM to adjust for the proportion of patients in this cohort who received adjuvant chemotherapy earlier, which was higher than in recent years. Another strength is the exclusion of patients who died within 6 months after diagnosis. This leads to more accurate associations between treatment and survival, as only patients who completed the period of treatment were included. This landmark analysis excluded 449 patients (5.7%) who died early, and the survival of the surgery only group especially increased as such, resulting in better representation of this patient group. Lastly, pathological tumour nodal staging was used instead of clinical tumour and nodal staging, enabling the selection of the appropriate patient group for potentially adjuvant chemotherapy. In the USA, for example, clinical staging is used to determine whether patients receive additional therapy after surgery, potentially overtreating patients who are downstaged after preoperative treatment.

Lastly, the distinction made between the three different neoadjuvant treatment groups in comparison with adjuvant treatment and no postoperative treatment constitutes a notable strength within the framework of this study. Previous research demonstrated an improved RFS only in patients treated with preoperative radiotherapy or no neoadjuvant treatment at all [31].

This study also has some limitations. Although we used PSM, information on performance status, comorbidities and type of adjuvant therapy for the patients was unfortunately not available. Patients who undergo adjuvant chemotherapy are usually in a relatively good clinical condition (ECOG) and have fewer comorbidities [32, 33]. Not taking into consideration the abovementioned elements could possibly provide a skewed overview of the clinical conditions of patients included in our cohort. An important nuance regarding the included population is that all patients underwent surgery, indicating they were at least sufficiently fit enough to successfully undergo TME. A second limitation concerning PSM is that we did not account for tumour location (distance to the anal verge). Recently, the definition of rectal cancer has changed because of wide global variation in the definition of the rectum [34]. Currently, the international consensus definition for the rectum is an anatomical landmark called the sigmoid take‐off as seen on a MRI or CT. Preoperative treatment schedules are different for rectal and sigmoid cancer and depend largely on these definitions. Unfortunately, research has shown that the sigmoid take‐off varies in distance from the anal verge amongst patients [35]. Thus, because of this change, some of the included rectal cancers could now be categorized as sigmoid cancer. The effect on OS of adjuvant chemotherapy has already been proven in sigmoid cancer, which could also partly account for the differences in OS [18, 19, 20].

Lastly, while our retrospective cohort study provides valuable insights, we acknowledge the presence of potential biases, particularly selection bias. Although we have attempted to mitigate these biases by using PSM, it is essential to interpret the results with an understanding of the inherent limitations.

## CONCLUSION

In this retrospective cohort study of a Dutch nationwide registry we conclude that patients with node‐positive rectal cancer, regardless of their preoperative treatment, could benefit from adjuvant chemotherapy in terms of 5‐year OS. Considering the previously mentioned limitations of the present study and the limited number of RCTs concerning adjuvant chemotherapy in patients with rectal cancer, further research is needed to establish the role of adjuvant chemotherapy in the multimodal treatment of rectal cancer.

## AUTHOR CONTRIBUTIONS

**Aaya Darai:** Methodology; investigation; data curation; formal analysis; visualization; writing – original draft; writing – review and editing. **Tijmen Koëter:** Conceptualization; methodology; data curation; validation; writing – review and editing. **Felice N. van Erning:** Writing – review and editing. **Robbert J. van Alphen:** Writing – review and editing. **Henk M. W. Verheul:** Writing – review and editing. **Marcel Verheij:** Writing – review and editing. **David D. E. Zimmerman:** Conceptualization; methodology; writing – review and editing. **Pauline A. J. Vissers:** Formal analysis; methodology; data curation; investigation; writing – review and editing. **Johannes H. W. de Wilt:** Conceptualization; methodology; supervision; validation; writing – review and editing; investigation.

## FUNDING INFORMATION

No funding.

## CONFLICT OF INTEREST STATEMENT

No conflict of interest to decline.

## ETHICS STATEMENT

The study was designed and reported in accordance with the STROBE checklist to ensure standardized reporting. This study was approved by the Privacy Review Board of the NCR and the scientific committee of the Prospective Dutch ColoRectal Cancer cohort. According to Dutch and European law the study did not require approval from an ethics committee in the Netherlands.

## Supporting information


**Data S1:** Supporting Information

## Data Availability

The data that support the findings of this study are available on request from the corresponding author. The data are not publicly available due to privacy or ethical restrictions.

## References

[1] Bender U , Rho YS , Barrera I , Aghajanyan S , Acoba J , Kavan P . Adjuvant therapy for stages II and III colon cancer: risk stratification, treatment duration, and future directions. Curr Oncol. 2019;26(Suppl 1):S43–s52. 31819709 10.3747/co.26.5605 PMC6878933

[2] Sargent D , Sobrero A , Grothey A , O'Connell MJ , Buyse M , Andre T , et al. Evidence for cure by adjuvant therapy in colon cancer: observations based on individual patient data from 20,898 patients on 18 randomized trials. J Clin Oncol. 2009;27(6):872–877. 19124803 10.1200/JCO.2008.19.5362 PMC2738431

[3] van Erning FN , Janssen‐Heijnen MLG , Creemers GJ , Pruijt JFM , Maas HAAM , Lemmens VEPP . Recurrence‐free and overall survival among elderly stage III colon cancer patients treated with CAPOX or capecitabine monotherapy. Int J Cancer. 2017;140(1):224–233. 27615021 10.1002/ijc.30423

[4] Grothey A , Sobrero AF , Shields AF , Yoshino T , Paul J , Taieb J , et al. Duration of adjuvant chemotherapy for stage III colon cancer. N Engl J Med. 2018;378(13):1177–1188. 10.1056/NEJMoa1713709 29590544 PMC6426127

[5] Bos AC , van Erning FN , Gestel YR , Creemers GJ , Punt CJ , van Oijen MG , et al. Timing of adjuvant chemotherapy and its relation to survival among patients with stage III colon cancer. Eur J Cancer. 2015;51(17):2553–2561. 10.1016/j.ejca.2015.08.016 26360411

[6] Des Guetz G , Nicolas P , Perret G‐Y , Morere J‐F , Uzzan B . Does delaying adjuvant chemotherapy after curative surgery for colorectal cancer impair survival? A meta‐analysis. Eur J Cancer. 2010;46(6):1049–1055. 20138505 10.1016/j.ejca.2010.01.020

[7] Paschke S , Jafarov S , Staib L , Kreuser ED , Maulbecker‐Armstrong C , Roitman M , et al. Are colon and rectal cancer two different tumor entities? A proposal to abandon the term colorectal cancer. Int J Mol Sci. 2018;19(9):2577. 30200215 10.3390/ijms19092577 PMC6165083

[8] Benson AB , Venook AP , al‐Hawary MM , Azad N , Chen YJ , Ciombor KK , et al. Rectal cancer, version 2.2022, NCCN clinical practice guidelines in oncology. J Natl Compr Cancer Netw. 2022;20(10):1139–1167. 10.6004/jnccn.2022.0051 36240850

[9] Wolpin BM , Mayer RJ . Systemic treatment of colorectal cancer. Gastroenterology. 2008;134(5):1296–1310. 18471507 10.1053/j.gastro.2008.02.098 PMC2528832

[10] Luzietti E , Pellino G , Nikolaou S , Qiu S , Mills S , Warren O , et al. Comparison of guidelines for the management of rectal cancer. BJS Open. 2018;2(6):433–451. 30511044 10.1002/bjs5.88 PMC6254003

[11] Petersen SH , Harling H , Kirkeby LT , Wille‐Jørgensen P , Mocellin S . Postoperative adjuvant chemotherapy in rectal cancer operated for cure. Cochrane Database Syst Rev. 2012;2012(3):CD004078. 22419291 10.1002/14651858.CD004078.pub2 PMC6599875

[12] Breugom AJ , Swets M , Bosset JF , Collette L , Sainato A , Cionini L , et al. Adjuvant chemotherapy after preoperative (chemo)radiotherapy and surgery for patients with rectal cancer: a systematic review and meta‐analysis of individual patient data. Lancet Oncol. 2015;16(2):200–207. 10.1016/S1470-2045(14)71199-4 25589192

[13] Dutch Guideline Colorectal Carcinoma. 2019. Assessed 29 Oct 2019.

[14] Keikes L , van Oijen MGH , Lemmens VEPP , Koopman M , Punt CJA . Evaluation of guideline adherence in colorectal cancer treatment in The Netherlands: a survey among medical oncologists by the Dutch colorectal cancer group. Clin Colorectal Cancer. 2018;17(1):58–64. 10.1016/j.clcc.2017.10.007 29157662

[15] Kwakman JJM , Bond MJG , Demichelis RM , Koopman M , Hompes R , Elferink MAG , et al. Adjuvant chemotherapy in patients with clinically node‐negative but pathologically node‐positive rectal cancer in The Netherlands: a retrospective analysis. Eur J Cancer. 2024;197:113466. 38061213 10.1016/j.ejca.2023.113466

[16] PALGA Impact door inzicht – For Researchers. Available from: https://www.palga.nl/en_GB/for‐researchers. Accessed 7 Mar 2025.

[17] Primaire behandeling rectumcarcinoom . 2009. Available from: https://richtlijnendatabase.nl/richtlijn/colorectaal_carcinoom_crc/primaire_behandeling_rectumcarcinoom_bij_crc.html. Accessed 29 Oct 2019.

[18] Andre T , Boni C , Navarro M , Tabernero J , Hickish T , Topham C , et al. Improved overall survival with oxaliplatin, fluorouracil, and leucovorin as adjuvant treatment in stage II or III colon cancer in the MOSAIC trial. J Clin Oncol. 2009;27(19):3109–3116. 19451431 10.1200/JCO.2008.20.6771

[19] Taal BG , Van Tinteren H , Zoetmulder FA , NACCP Group . Adjuvant 5FU plus levamisole in colonic or rectal cancer: improved survival in stage II and III. Br J Cancer. 2001;85(10):1437–1443. 10.1054/bjoc.2001.2117 11720425 PMC2363941

[20] Moertel CG , Fleming TR , Macdonald JS , Haller DG , Laurie JA , Goodman PJ , et al. Levamisole and fluorouracil for adjuvant therapy of resected colon carcinoma. N Engl J Med. 1990;322(6):352–358. 2300087 10.1056/NEJM199002083220602

[21] Bosset JF , Calais G , Mineur L , Maingon P , Stojanovic‐Rundic S , Bensadoun R‐J , et al. Fluorouracil‐based adjuvant chemotherapy after preoperative chemoradiotherapy in rectal cancer: long‐term results of the EORTC 22921 randomised study. Lancet Oncol. 2014;15(2):184–190. 24440473 10.1016/S1470-2045(13)70599-0

[22] Breugom AJ , van Gijn W , Muller EW , Berglund Å , van den Broek CBM , Fokstuen T , et al. Adjuvant chemotherapy for rectal cancer patients treated with preoperative (chemo)radiotherapy and total mesorectal excision: a Dutch colorectal cancer group (DCCG) randomized phase III trial. Ann Oncol. 2015;26(4):696–701. 25480874 10.1093/annonc/mdu560

[23] Sainato A , Cernusco Luna Nunzia V , Valentini V , De Paoli A , Maurizi ER , Lupattelli M , et al. No benefit of adjuvant fluorouracil leucovorin chemotherapy after neoadjuvant chemoradiotherapy in locally advanced cancer of the rectum (LARC): long term results of a randomized trial (I‐CNR‐RT). Radiother Oncol. 2014;113(2):223–229. 10.1016/j.radonc.2014.10.006 25454175

[24] Bosset J‐F , Collette L , Calais G , Mineur L , Maingon P , Radosevic‐Jelic L , et al. Chemotherapy with preoperative radiotherapy in rectal cancer. N Engl J Med. 2006;355(11):1114–1123. 16971718 10.1056/NEJMoa060829

[25] Glynne‐Jones R , Counsell N , Quirke P , Mortensen N , Maraveyas A , Meadows HM , et al. Chronicle: results of a randomised phase III trial in locally advanced rectal cancer after neoadjuvant chemoradiation randomising postoperative adjuvant capecitabine plus oxaliplatin (XELOX) versus control. Ann Oncol. 2014;25(7):1356–1362. 24718885 10.1093/annonc/mdu147

[26] Nilsson PJ , van Etten B , Hospers GAP , Påhlman L , van de Velde CJH , Beets‐Tan RGH , et al. Short‐course radiotherapy followed by neo‐adjuvant chemotherapy in locally advanced rectal cancer—the RAPIDO trial. BMC Cancer. 2013;13:279. 23742033 10.1186/1471-2407-13-279 PMC3680047

[27] Glynne‐Jones R , Wyrwicz L , Tiret E , Brown G , Rödel C , Cervantes A , et al. Rectal cancer: ESMO clinical practice guidelines for diagnosis, treatment and follow‐up. Ann Oncol. 2018;29(Suppl 4):iv263. 29741565 10.1093/annonc/mdy161

[28] Snijders HS , Wouters MWJM , van Leersum NJ , Kolfschoten NE , Henneman D , de Vries AC , et al. Meta‐analysis of the risk for anastomotic leakage, the postoperative mortality caused by leakage in relation to the overall postoperative mortality. Eur J Surg Oncol. 2012;38(11):1013–1019. 22954525 10.1016/j.ejso.2012.07.111

[29] Wang S , Liu J , Wang S , Zhao H , Ge S , Wang W . Adverse effects of anastomotic leakage on local recurrence and survival after curative anterior resection for rectal cancer: a systematic review and meta‐analysis. World J Surg. 2017;41(1):277–284. 27743072 10.1007/s00268-016-3761-1 PMC5209428

[30] Arron MNN , Greijdanus NG , Bastiaans S , Vissers PAJ , Verhoeven RHA , Ten Broek RPG , et al. Long‐term oncological outcomes after colorectal anastomotic leakage: a retrospective Dutch population‐based study. Ann Surg. 2022;276(5):882–889. 10.1097/SLA.0000000000005647 35930021 PMC9534056

[31] van Erning FN , Rutten HJT , van den Berg HA , Lemmens VEPP , van Halteren HK . Effect of adjuvant chemotherapy on recurrence‐free survival varies by neo‐adjuvant treatment in patients with stage III rectal cancer. Eur J Surg Oncol. 2015;41(12):1630–1635. 26437853 10.1016/j.ejso.2015.09.011

[32] Watanabe A , Yang C , Cheung WY . ECOG performance status as a predictor of adjuvant chemotherapy (AC) toxicities in stage III colorectal cancer (CRC) patients. J Clin Oncol. 2017;35(4_suppl):789. 10.1200/JCO.2017.35.4_suppl.789

[33] Rocha L , Riechelmann RP . Treatment of patients with metastatic colorectal cancer and poor performance status: current evidence and challenges. Clinics (Sao Paulo). 2018;73(suppl 1):e542s. 30281700 10.6061/clinics/2018/e542s PMC6142860

[34] D'Souza N , tot Babberich MPMN , d'Hoore A , Tiret E , Xynos E , Beets‐Tan R , et al. Definition of the rectum: an international, expert‐based Delphi consensus. Ann Surg. 2019;270(6):955–959. 30973385 10.1097/SLA.0000000000003251

[35] D'Souza N , Balyasnikova S , Tudyka V , Lord A , Shaw A , Abulafi M , et al. Variation in landmarks for the rectum: an MRI study. Colorectal Dis. 2018;20(10):O304–O309. 30176118 10.1111/codi.14398

