# Supplementary material for: The role of adjuvant chemotherapy in rectal cancer: A nationwide cohort study from the Netherlands
Source: Colorectal Dis. 2025 Mar 9;27(3):e70054. doi: 10.1111/codi.70054 (PMC11891379; doi:10.1111/codi.70054)
Supplement: Supplementary file 1 — Data S1: Supporting Information [file CODI-27-0-s001.docx]

**Supplementary data**

**Table 1**

Patient-, tumour, and treatment characteristics of the total study population.

|  | Group | No adjuvant systemic therapy | Adjuvant systemic therapy | Standardized mean differences |
| --- | --- | --- | --- | --- |
|  | Total patients | 6614 | 865 |  |
|  |  | N (%) | N (%) |  |
| **Patient** |  |  |  |  |
| Age |  |  |  | SMD = 0.484 |
|  | < 60 yr | 1851 (28.0) | 387 (44.7) |  |
|  | 61-70 yrs | 2244 (33.9) | 326 (37.7) |  |
|  | 71-80 yrs | 1901 (28.7) | 145 (16.8) |  |
|  | >80yrs | 618 (9.3) | 7 (0,8) |  |
| Sex |  |  |  | SMD = 0.021 |
|  | Male | 4112 (62.2) | 546 (63.1) |  |
|  | Female | 2502 (37.8) | 319 (36.9) |  |
| Year of diagnosis |  |  |  | SMD = 0.703 |
|  | 2009-2012 | 1646 (24.9) | 510 (59) |  |
|  | 2013-2016 | 2780 (42.0) | 234 (27.1) |  |
|  | 2017-2020 | 2188 (33.1) | 121 (14.0) |  |
| **Tumour** |  |  |  |  |
| (y)pT stage |  |  |  | SMD = -0.152 |
|  | (y)pT0 – (y)pT2 | 2127 (32.2) | 230 (24.6) |  |
|  | (y)pT3 | 4178 (63.2) | 561 (64.9) |  |
|  | (y)pT4 | 309 (4.7) | 74 (8.6) |  |
| (y)pN stage |  |  |  | SMD =-0.369 |
|  | (y)pN1 | 5034 (76.1) | 515 (59.5) |  |
|  | (y)pN2 | 1580 (23.9) | 350 (40.5) |  |
| **Treatment** |  |  |  |  |
| Type of surgery |  |  |  | SMD = 0.323 |
|  | (Low) anterior resection | 4820 (72.9) | 752 (86.9) |  |
|  | Abdominoperineal resection | 1794 (27.1) | 113 (13.1) |  |
| Neoadjuvant |  |  |  | SMD = 0.319 |
|  | Radiotherapy | 2664 (40.3) | 356 (41.2) |  |
|  | Chemoradiotherapy | 2303 (34.8) | 190 (22.0) |  |
|  | None | 1647 (24.9) | 319 (36.9) |  |
| Anastomotic complications |  |  |  | SMD = 0.493 |
|  | No leakage or abscess | 4285 (64.8) | 742 (85.8) |  |
|  | Leakage and/or abscess | 774 (11.7) | 81 (9.4) |  |
|  | Not applicable* | 1555 (23.5) | 42 (4.9) |  |

* Not applicable due to having a deviating stoma.

**Patients with rectal cancer**

**N=8965**

**Patients N=7928**

**Patients N=7479**

**Patients N=7945**

**Unknown complications**

**N = 468**

**Patients N=8413**

**No APR or LAR**

**N = 245**

**Metastatic cancer**

**N =74**

**pTumorstage (p0/pX) N=233**

N =233

**Patients N=8658**

**Patients N=8732**

**Only neoadjuvant chemotherapy
N=17**

**N = 17**

**landmark analysis 6 months
N = 449**

**Flowchart 1** patient inclusion
